# Are Anxiety Disorders Associated with Accelerated Aging? A Focus on Neuroprogression

**DOI:** 10.1155/2016/8457612

**Published:** 2015-12-31

**Authors:** Giampaolo Perna, Giuseppe Iannone, Alessandra Alciati, Daniela Caldirola

**Affiliations:** ^1^Department of Clinical Neurosciences, Hermanas Hospitalarias, FoRiPsi, Villa San Benedetto Menni, Albese con Cassano, 22032 Como, Italy; ^2^Department of Psychiatry and Neuropsychology, Faculty of Health, Medicine and Life Sciences, Maastricht University, 6200 MD Maastricht, Netherlands; ^3^Department of Psychiatry and Behavioral Sciences, Leonard Miller School of Medicine, Miami University, Miami, FL 33136, USA

## Abstract

Anxiety disorders (AnxDs) are highly prevalent throughout the lifespan, with detrimental effects on daily-life functioning, somatic health, and quality of life. An emerging perspective suggested that AnxDs may be associated with accelerated aging. In this paper, we explored the association between AnxDs and hallmarks of accelerated aging, with a specific focus on neuroprogression. We reviewed animal and human findings that suggest an overlap between processes of impaired neurogenesis, neurodegeneration, structural, functional, molecular, and cellular modifications in AnxDs, and aging. Although this research is at an early stage, our review suggests a link between anxiety and accelerated aging across multiple processes involved in neuroprogression. Brain structural and functional changes that accompany normal aging were more pronounced in subjects with AnxDs than in coevals without AnxDs, including reduced grey matter density, white matter alterations, impaired functional connectivity of large-scale brain networks, and poorer cognitive performance. Similarly, molecular correlates of brain aging, including telomere shortening, A*β* accumulation, and immune-inflammatory and oxidative/nitrosative stress, were overrepresented in anxious subjects. No conclusions about causality or directionality between anxiety and accelerated aging can be drawn. Potential mechanisms of this association, limitations of the current research, and implications for treatments and future studies are discussed.

## 1. Introduction

Anxiety disorders (AnxDs) are highly prevalent across the lifespan in the general population. Pooled 1-year and lifetime prevalence have been estimated at around 11% and 17%, respectively [1]. Different AnxDs are more prevalent at specific lifespan stages. Phobias predominate in childhood, panic disorder (PD) predominates in adulthood, and generalized anxiety disorder (GAD) and agoraphobia (AG) predominate in adulthood and older age. AnxDs can also have a late onset, with an incidence of 3-4% after 55–60 years of age [2–4].

AnxDs are chronic and stressful conditions that can negatively affect quality of life, somatic health, and cognitive performance. Several studies documented that anxiety is a risk factor for many age-related medical conditions, such as coronary heart disease, diabetes, and disability, as well as for global mortality [5–7]. Recent findings showed an association between AnxDs or anxiety symptoms and reduced verbal memory, language, and executive functions in older individuals without dementia [8–11].

An emerging perspective suggested that in people with AnxDs decreased somatic health or cognition may partly result from accelerated cellular aging and neuroprogression. Neuroprogression is pathological reorganization of the central nervous system (CNS), along the course of severe mental disorders, leading to cerebral structural changes and functional alterations. It is a combination of increased neurodegeneration, neuronal apoptosis or neurotoxic susceptibility, and lowered neuroplasticity [12]. Neuroplasticity refers to the ability of the brain to modify itself in response to environmental demands and it plays an important role in optimizing brain functionality. It encompasses neurogenesis, structural and functional brain reorganization, cellular and molecular changes, and cognitive plasticity [13]. These processes occur throughout the lifespan in response to a wide array of genetic and environmental factors. Neuroplasticity is downregulated in adulthood and old age and its impairment can negatively impact successful aging [14] and cognitive performance [15]. Neuroprogression has been extensively investigated in major depressive disorder (MDD)/bipolar disorder (BD) and several potential mechanisms of neuroprogression have been proposed, including immune-inflammatory and oxidative/nitrosative stress with its concomitants and sequels, dysregulation of the hypothalamic-pituitary-adrenal (HPA) axis, autonomic nervous system (ANS), and immune system or neurotransmitters' functioning (for detailed reviews, see [12, 16–19]). This research on AnxDs is at the early stage. However, some neuroprogressive pathways found in MDD/BD may be present also in subjects with AnxDs and contribute to accelerated aging and neuroprogression in this population [20].

In this paper, we reviewed evidence of an association between AnxDs, according to DSM-5 criteria [21], and hallmarks of accelerated aging, with a focus on neuroprogression. Thus, we explored, in animal and human studies, the overlap between processes of neurogenesis, neurodegeneration, structural, functional, molecular, and cellular modifications in AnxDs, and aging. To the best of our knowledge, no reviews on this issue have been published.

## 2. Materials and Methods

This is a nonsystematic review. Data were sourced from PubMed electronic database and were not limited by date of publication. Only articles written in English language were considered.

## 3. Neurogenesis 

Neurogenesis refers to the formation, growth, and development of new neurons from neural stem cells and progenitor cells. Adult neurogenesis in humans is restricted to the hippocampus (subgranular and subventricular zones of the dentate gyrus) [22–24].

### 3.1. Impaired Neurogenesis in Anxiety

Adult hippocampal neurogenesis (AHN) is impaired in rodent models of anxiety, including chronic unpredictable mild stress, repeat restraint stress, social defeat stress, and corticosterone administration, as well as in models of social stress in nonhuman primates, such as the intruder stress and social isolation models. These paradigms trigger anxiety- and depression-like behaviors in animals, suggesting a possible association between both anxiety and depression and altered AHN [25]. In rodent models of childhood neglect (which is a risk factor for future anxiety and mood disorders in humans), young rats separated from their mothers exhibited both increased anxiety and decreased AHN in adulthood [26]. Recently, decreased hippocampal number of neuroblasts and dendritic arborization related to high corticosterone were found in Carioca High-Conditioned Freezing rats, an animal model of generalized anxiety disorder (GAD) [27]. Transgenic mice in which AHN was impaired exhibited significant increased anxiety-like behavior [28, 29]. Finally, in rodents, disrupting AHN negatively affected pattern separation, which is the learning process by which similar experiences are transformed into distinct, nonoverlapping representations [30]. Since pattern separation impairment seems to be implicated in overgeneralization of conditioned fear in AnxDs, an association between reduced AHN and anxiety may exist in humans [31]. Both in stressed rodents [32] and in nonhuman primates, antidepressants, which are the first-line treatment for AnxDs, increase AHN which, in turn, can diminish anxiety-like behavior [33–35].

No published postmortem brains studies have directly or indirectly measured AHN in humans with AnxDs. High-resolution MRI volumetric studies showed smaller dentate gyrus size in subjects with anxiety [25], but the extent to which this may be related to changes in AHN or to other forms of structural plasticity remains to be determined. Finally, no studies are available on the relationship between medications and AHN in individuals with AnxDs.

In summary, animal models showed that altered neurogenesis may be associated with anxiety, but whether accelerated AHN impairment is also related to human anxiety remains an open issue. In clinical samples, direct AHN assessment is needed, and new noninvasive measurements of AHN in humans, such as by SPECT or MRI, are emerging [36, 37]. In light of preclinical data and given that multiple biological alterations in people with AnxDs, including higher levels of corticosteroids [38], and proinflammatory factors [39] and/or lower levels of growth factors [40] have well-known detrimental effects on AHN [41, 42], this field is worth of being further investigated.

### 3.2. Impaired Neurogenesis in Aging

In animal studies, aging has been associated with significant decline in adult hippocampal neurogenesis (AHN) in rodents [43, 44], canines [45], and marmosets [46]. Several studies showed that AHN in rats decreases by 80% by about one-two years of age [47–49]. Also, in humans, the formation of new neurons is abundant during infancy and adolescence and dramatically decreases during adulthood and especially in old age. Although decreased neurogenesis may exert important protective effects, such as tumor prevention [50], it seems also to be linked to cognitive flexibility impairment in mice [51] and age-related cognitive deficits in humans [52].

In conclusion, preliminary evidence suggests that anxiety may be associated with decreased neurogenesis, similar to what has been observed during aging.

## 4. Brain Structural Changes 

### 4.1. Brain Structural Changes in Anxiety

In murine models, hippocampal volume and trait anxiety were inversely related [53], and stress-related hypercortisolemia or chronic treatment with corticosterone resulted in hippocampal atrophy and anxiety-like behaviors [54, 55]. In nonhuman primates, high trait-like anxiety has been associated with smaller volume of the dorsal anterior cingulate cortex (dACC), which is a portion of the prefrontal cortex (PFC) [56]. In humans, several structural neuroimaging studies compared people with AnxDs to healthy controls. In subjects with panic disorder (PD), reduced volume of the temporal lobe, as well as reduced gray matter (GM) density in the amygdala and hippocampus, was found. GM abnormalities have also been found in the bilateral putamen, left orbitofrontal cortex, inferior frontal cortex, superior temporal gyrus, right insula, and anterior cingulate cortex [57, 58]. In GAD, decreased structural connectivity between the amygdala, the anterior cingulate cortex (ACC), and the PFC was found. Other studies showed reduced hippocampal volume, decreased white matter (WM) in the ACC and middle cingulated cortex integrity, and decreased GM volumes in the precentral gyrus, precuneus, orbitofrontal gyrus, and posterior cingulate gyrus [59]. Disrupted WM microstructure coherence of the right splenium and right parietal cortex was also found [60]. Finally, preliminary investigation showed altered structural brain connectivity in patients with social anxiety disorder (SAD) suggesting frontal WM alteration in or near the uncinate fasciculus, a structure that connects anterior temporal areas with prefrontal/orbitofrontal cortices [61].

### 4.2. Brain Structural Changes in Aging

Brain structural alterations accompany normal aging. SAMP10 mice, a strain of inbred mice developed to study human aging, exhibited age-related cortical atrophy in the frontal cortex, occipital lobes, olfactory bulbs, amygdala, and entorhinal cortex [62]. In humans, postmortem and structural neuroimaging findings showed age-related brain atrophy (0.4–0.5% brain tissue loss per year), as indicated by reduced brain volume and weight, ventricular expansion, and sulcal enlargement [63]. Prominent age-related GM loss has been demonstrated both cross-sectionally and longitudinally in the frontal and prefrontal areas, hippocampus, temporal and parietal cortices, amygdala, and cerebellum and was accompanied by shrinkage and dysmorphology of neurons and deafferentation and reduction in synaptic density [64–69]. Structural WM degeneration occurs in the entire brain and mainly in the frontal cortex [70, 71]. Both GM and WM structural alterations are likely to impair communication within and between brain areas and lead to age-related cognitive decline [72].

In conclusion, anxiety has been associated with several brain structural changes, some of which are similar to those observed during aging.

## 5. Brain Functional Changes

Functional connectivity reflects the quality of information transfer and functional communication between brain areas that increase or decrease their activity synchronically. Among these, a network of brain regions plays a relevant role during resting states: the default-mode network (DMN) (i.e., the “task-negative” network) that consists of the precuneus/posterior cingulate cortex (PCC), medial prefrontal cortex, medial temporal regions, medial, lateral, and inferior parietal cortex, and portions of the ACC, and it is active during internally directed mental states, such as introspective states, remembering, planning, and related cognitive functions, and emotion regulation. The DMN is connected with the “task-positive” network that consists of the dorsolateral prefrontal cortex, inferior parietal cortex, and supplementary motor area and it is associated with task-related patterns of increased attentional orientation and response preparation [73].

### 5.1. Brain Functional Changes in Anxiety

Impaired functioning of several brain networks involved in cognition and motivation has been found in subjects with anxiety. Individuals with different AnxDs (in particular GAD and SAD) or with high trait anxiety presented decreased functional connectivity among areas of the cinguloopercular and frontoparietal networks compared to controls, resulting in impaired detecting errors/conflicts and cognitive control to resolve future conflicts. Functional changes within the frontoparietal network and between the cinguloopercular and frontoparietal networks and the amygdala were also found [74]. Decreased functioning of the DMN [74] and its functional connectivity with the amygdala has been observed in subjects with AnxDs compared to healthy controls [75, 76]. A recent study comparing subjects with GAD and healthy controls indicated that the presence of GAD, longer duration of illness, and symptoms severity exacerbated the effects of age on decreased functional connectivity in the DMN, in particular between the posterior cingulate and the medial prefrontal cortex and between the PCC and the medial prefrontal cortex [77].

### 5.2. Brain Functional Changes in Aging

Normal aging is characterized by disrupted coordination of these large-scale brain systems, which may be partly responsible for the cognitive decline during aging. These brain regions are particularly vulnerable to atrophy and amyloid deposition [78]. Poorer cognitive performance in the elderly seems to be a consequence of both increased lateralized intranetwork and decreased internetwork connectivity, which may result in more diffuse and less specialized patterns of functional connections that negatively impact cognition [79–82]. Indeed, in healthy older individuals, several brain imaging studies showed decreased functional connectivity across several regions of the DMN, both at rest and during cognitive tasks, which was associated with impaired performance in processing speed, memory, and executive functions [83–86].

In conclusion, preliminary evidence suggests an association between anxiety and impaired functional connectivity, similar to what has been found during aging.

## 6. Cognitive Decline 

### 6.1. Cognitive Decline in Anxiety

Both animal and human studies suggested that anxiety may be associated with cognitive changes similar to those observed during normal aging. In mutant mice, anxiety correlated with impaired spatial learning and memory [87]. Transgenic mice with higher levels of corticosterone (an animal model reproducing hyperactivity of the HPA axis, which is often seen in AnxDs) exhibited learning and memory plasticity deficits [88]. In tree shrews stressful experiences increasing cortisol levels resulted in declarative memory deficits that persisted several weeks afterward despite rebound cortisol levels [89]. Neuropsychological studies on individuals with AnxDs yielded mixed results, probably because of different sampling, methodology, neuropsychological test batteries, and lack of control for confounding variables, such as pharmacological treatments. Preliminary findings suggested that subjects with GAD have poorer performance in processing speed, verbal memory, working memory, cognitive flexibility, and executive functions compared to healthy controls [90–92]. Individuals with PD or SAD exhibited poorer verbal memory, attention, learning, and executive functions [93–98]. In late-life major depressive disorder comorbid AnxDs were associated with greater memory decline at 4-year follow-up [99]. In a sample of older individuals without dementia, anxiety symptoms were associated with memory loss and predicted both cognitive decline and daily-life functioning impairment after 3 years [100]. Anxiety symptoms occurred more frequently in persons with mild cognitive impairment (MCI) than in cognitively intact elderly individuals from the general population and significantly increased risk of progression from MCI to Alzheimer's disease at 3-year follow-up [101]. Finally, in a prospective cohort study of individuals aged 65 to 96 years, incident cognitive impairment was associated with baseline AnxDs in men and with anxiety symptoms in women, independently of depression [102].

### 6.2. Cognitive Decline in Aging

Advanced aging is accompanied by cognitive decline that is related to structural and functional changes [103]. In healthy older individuals, maintenance of both higher cortical volume and WM complexity has been associated with successful cognitive performance. In the elderly, strong correlations emerged between hippocampal volume and global cognition and memory, between frontal areas volume and executive function [104, 105], and between WM complexity and information processing speed, auditory-verbal learning, and reasoning [106]. Reduced mental speed [107], executive function [108], and episodic memory [109] were found whereas verbal ability and word knowledge were often maintained [110]. As previously described, diminished functional connectivity across several regions of DMN during aging is associated with progressive cognitive decline in several cognitive domains, including attention, concentration, processing speed, memory, and executive functioning [83–85]. The age-related reduced ability to decrease DMN activity when attention is required seems particularly relevant to cognitive and goal-directed activity impairment [111].

In conclusion, AnxDs and aging seem to share reduced cognitive abilities that may be related to the similar structural and/or functional brain changes described above.

## 7. Beta-Amyloids 

Beta-amyloids (A*β*) are protein fragments implicated in neurodegeneration, cellular aging, and cognitive deterioration [112, 113]. At high concentrations, A*β* can negatively influence AHN [114], synaptic functions, and monoaminergic transmission and can have cytotoxic effects and functional antagonism with brain-derived neurotrophic factor (BDNF) [112, 113].

### 7.1. Beta-Amyloids in Anxiety

In animal studies, a relationship between anxiety and A*β* levels was found. Stress-level glucocorticoids administration in mice increased A*β* production and augmented tau accumulation, suggesting that glucocorticoids, which are also implicated in human AnxDs, may be related to A*β* pathology and development of neurofibrillary tangles (i.e., two neuropathological hallmarks of Alzheimer's disease and severe cognitive decline) [115]. Similarly, behavioral stressors (social isolation over 3 months or acute restraint stress) increased A*β* levels in the brain interstitial fluid, hippocampus, and cortex of mice via corticotropin-releasing factor [116]. Cerebral injection of A*β* fragment in rats exerted profound negative effects on the hippocampus and amygdala and induced both anxiety-like behaviors and memory impairment [117, 118].

Human research on this topic is scant. In middle-aged and older nondemented adults, a PET study found significant associations between trait anxiety symptoms and amyloid senile plaques and tau neurofibrillary tangles in the posterior cingulate of subjects with mild cognitive impairment (MCI) and in the medial temporal and frontal areas of subjects with no cognitive deficits [119]. In subjects with MCI, a significant association was found between A*β*42 and t-tau abnormal concentrations in the cerebrospinal fluid and anxiety symptoms severity [120]. Finally, in a prospective cohort of healthy older adults, anxiety symptoms seem to moderate, with a dose-effect relationship, the negative effects of A*β* on global cognition, resulting in more rapid decline in several cognitive domains [121, 122].

### 7.2. Beta-Amyloids in Aging

In rhesus monkeys, a significant age-related A*β* increase was found in the basal forebrain cholinergic neurons [123]. Human PET studies showed that about one-third of healthy elderly individuals manifested elevated levels of A*β* deposition in the frontal, cingulated, and parietal areas and in the DMN, even years before clinical cognitive deficits [124, 125]. While some studies failed to report significant associations between amyloid deposition and cognitive decline [126], others found that greater amyloid deposition was negatively related to episodic memory performance and decline in healthy older adults [127–131]. A very recent study [132] showed that normal elderly individuals with high A*β* plasma levels presented lower cognitive performance and thinner cortex than those with low A*β* levels.

In conclusion, the available findings suggest an association between anxiety and A*β* pathology and indicate that this is a critical topic worth of future investigation.

## 8. Telomere Shortening 

Telomeres are specialized DNA-protein complexes found at the ends of chromosomes. Small portions of telomeric DNA are normally lost with time and cell division: when telomeres get too short, the cell can no longer divide and eventually dies. Telomere shortening is progressive with age and is considered a biomarker of cellular aging/damage and disease [133].

### 8.1. Telomere Shortening in Anxiety

Both animal and human findings showed an association between anxiety and telomere shortening. Deficiency of telomerase (i.e., an enzyme that preserves telomere length by adding telomeric DNA) resulted in increased anxiety-like behavior in aged transgenic mice when compared with wild-type mice [134]. In human nonpsychiatric samples, associations were found between exposure to chronic stress (e.g., childhood adverse experiences/stressful caregiving status) or high phobic anxiety and accelerated telomere shortening, which may be related to dysregulation of inflammatory markers, HPA axis, and autonomic system function [135–138]. Longitudinal findings demonstrated that AnxDs predicted shorter leukocytes telomeres at 2 years of follow-up in the general population, whereas depressive disorders did not [139], and persistence of internalizing psychiatric disorders, including GAD, from adolescence to adulthood, predicted shorter telomere length at age 38 [140]. Patients with current AnxDs, but not remitted, had shorter leukocyte telomeres compared to healthy controls [20], suggesting that telomere shortening may be partly reversible. Furthermore, anxiety symptoms severity was associated with telomere shortening in the whole sample, suggesting a dose-response association, similar to what was found by Okereke and coworkers [138]. Young women with GAD or PD had shorter telomeres than women with no GAD or PD [141] and older subjects with AnxDs had significantly shorter telomeres than coeval healthy controls [142], suggesting that anxiety may accelerate age-related telomere shortening.

### 8.2. Telomere Shortening in Aging

Telomere shortening increases with age [133]. Preclinical studies demonstrated that insufficient telomerase activity impairs telomere length restoration, enhancing susceptibility to cellular senescence and death [143]. In adult and old mice with critically short telomeres, dietary supplementation of the telomerase activator TA-65 increased average telomere length and improved many health-span indicators [144].

Human studies also point to a causal relationship between telomere shortening and increased risk of age-related disease, including cancer, diabetes, and coronary heart disease [143, 145, 146]. Since cell or tissue dysfunction is triggered by severe telomere shortening, telomerase activation may promote health maintenance. In humans telomere shortening can be also delayed by telomerase activator dietary supplementation [147] which enhanced several indicators of metabolic, bone, and cardiovascular health (e.g., glycemia, cholesterol, and blood pressure) at 5-year follow-up [148]. Telomeres seem to be involved in neurodegeneration and neurodegenerative diseases as well. Molecular mechanisms of neurodegeneration, such as abnormal levels of A*β*, may accelerate neuronal senescence through telomere attrition [149]. An association between shorter telomeres and poorer cognitive performance has been observed in general elderly populations, suggesting that telomere length may serve as a biomarker of cognitive aging [150, 151]. Telomere shortening is modulated by both genetic and nongenetic factors, including oxidative stress, inflammation, physical activity, and lifestyle [150, 151].

In conclusion, anxiety may be related to shorter telomeres which also characterizes aging and age-related diseases and cognitive decline.

## 9. Activated Immune-Inflammatory Pathways

**Figure 1:**
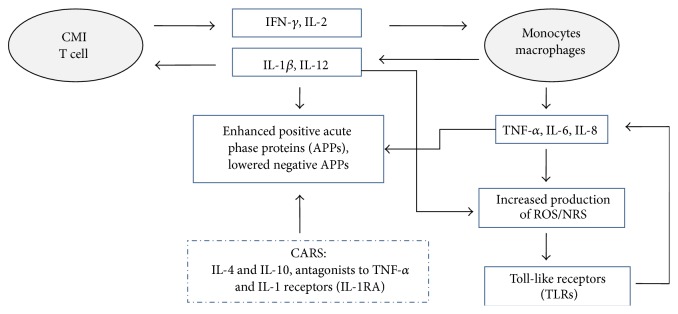
Relationship between activated immune-inflammatory pathways and oxidative/nitrosative stress (O&NS). CMI involves activation of T cells that produce cytokines such as IFN-*γ* and IL-2, which activate monocytes/macrophages. Monocytes/macrophages produce IL-1*β* and IL-12 (that exert a positive feedback loop on T cells), as well as TNF-*α*, IL-6, and IL-8. Proinflammatory macrophage-derived cytokines (PICs) mediate inflammation enhancing the positive acute phase proteins (APPs), for example, C-reactive protein, and lowering the negative APPs, for example, albumin. The counterinflammatory response syndrome (CARS) tends to dampen the acute inflammatory response producing IL-4 and IL-10 (responsible for decreasing TNF-*α*, IL-1, IL-6, and IL-8) and the antagonists to TNF-*α* and IL-1 receptors (IL-1RA), which inactivate the cytokine or block the receptors. Immune inflammation and O&NS influence each other. Inflammatory and CMI responses are accompanied by increased production of reactive oxygen species (ROS) and reactive nitrogen species (RNS) while oxidative stress maintains inflammation mainly through the activation of toll-like receptors (TLRs). Damaged macromolecules released during condition of oxidative stress can activate TLRs which produce an inflammatory response whose key mediators are IL-1, IL6, and TNF-*α*. CMI: cell-mediated immune; IL-6: interleukin-6; IL-1*β*: interleukin-1*β*; IL-12: interleukin-12; TNF-*α*: tumor necrosis factor-*α*; IFN-*γ*: interferon-*γ*; APPs: acute phase proteins; TLRs: toll-like receptors.

Activated immune-inflammatory pathways are considered “core” components of neuroprogressive changes [17]. Cell-mediated immunity (CMI) involves activation of T cells that produce cytokines such as IFN-*γ* and IL-2, which activate monocytes/macrophages. In turn, monocytes/macrophages produce several cytokines such as IL-1*β* (exerting a positive feedback loop on T cells), IL-12 (triggering T cells to produce more IFN-*γ*), TNF-*α*, IL-6, and IL-8. Inflammation consists of cellular, cytokine, and complement cascades and an acute phase response. Macrophage-derived cytokines, known as proinflammatory cytokines (PICs), mediate inflammation by enhancing the positive acute phase proteins (APPs), for example, C-reactive protein (CRP) and haptoglobin, and lowering the negative APPs, for example, albumin and transferrin. During inflammation, also counter-anti-inflammatory mechanisms become active (e.g., increased production of the IL-1 receptor antagonist) to dampen the primary inflammatory response [12, 152]. Activated immune-inflammatory pathways increase oxidative/nitrosative processes [153] (Figure 1).

### 9.1. Activated Immune-Inflammatory Pathways in Anxiety

Preclinical and human studies suggested that anxiety is associated with CMI activation and inflammation. Although results are mixed [154], some animal studies showed a relationship between increased proinflammatory cytokines levels including interleukin-6 [155] and IL-1*β* [156] and anxiety-like behaviors. In mice, sustained inflammatory pain, with concomitant TNF-*α* increase in basolateral amygdala, was associated with anxiety-like behaviours which was reversed by local infusion of infliximab, a TNF-*α* neutralizing antibody [157]. In humans, significantly increased levels of proinflammatory cytokines have been detected in patients with AnxDs compared to nonanxious subjects, independently of sociodemographic features and depressive symptoms [39, 158]. Higher inflammatory dysregulation was especially found in persons with late-onset AnxDs [159]. Recently, PD has been associated with lower levels of mannan-binding lectin (MBL), an important arm of the innate immune system, the deficiency of which may result in infections or autoimmune diseases [160]. Plasma anti-serotonin and serotonin anti-idiotypic antibodies are elevated in PD compared to healthy controls, suggesting a link between autoimmune mechanisms and AnxDs [161]. In the general population, anxiety symptoms were associated with increase of several inflammation markers, including C-reactive protein, TNF-*α*, and IL-6, even after adjusting for multiple confounding factors [162].

Activated immune inflammation may be related to anxiety also through its influence on serotoninergic pathways. During CMI activation, cytokines, mainly IFN-*γ*, induce indoleamine 2,3-dioxygenase (IDO) [163] which, in turn, stimulates the catabolism of tryptophan leading to its plasma depletion and synthesis of tryptophan catabolites (TRYCATs). The TRYCATs kynurenine and quinolinic acid induced anxiety-like behaviours in animal models [164]. In humans, a correlation between plasma kynurenine concentration and caffeine-induced anxiety has been found [165]. Several studies suggested a relationship between activated immune- inflammatory pathways and increased intestinal permeability, called leaky gut [166]. It is characterized by the weakening of the tight junctions' barrier, formed by epithelial cells, which segregates the luminal bacteria in the gut, and can be produced by inflammatory processes [167] and/or by oxidative stress [168]. When leaky gut is present, Gram-negative bacteria or lipopolysaccharide (LPS), a component of the outer membrane of Gram-negative bacteria, is translocated from the gut to mesenteric lymphonodes and, consequently, CMI activation with cytokines release may be elicited. Gut-derived bacterial products, such as LPS, can induce anxiety-related behaviors (e.g., reduced exploratory behavior and social interactions) when administered to rodents [169–171] and cause acute anxiety and cognitive deficits in healthy male volunteers [172]. In line with this, recent animal studies showed that diet-induced changes in the gut microbiota influence long- and short-term memory and cognitive flexibility in mice [173]. LPS effects could partly be related to the LPS-induced elevation of peripheral cytokine that, in turn, may affect amygdala activity [174].

Moreover, LPS-induced inflammation can enhance the IDO activity and the availability of kynurenine, which has been shown to increase anxiety when administered peripherally to mice [175]. Data on TRYCATs and leaky gut/LPS pathways in subjects with AnxDs are still lacking and future studies are warranted.

### 9.2. Activated Immune-Inflammatory Pathways in Aging

“Inflammaging” refers to the chronic progressive inflammatory status of the brain during aging [176]. In mice, TREM2 expression (an immune receptor involved in suppressing inflammatory responses) increased during aging [177] and protected against aging-related neuroinflammation, neuronal losses, and cognitive impairment [178]. Human investigations showed that elderly people exhibit chronically increased levels of proinflammatory cytokines and reduced levels of anti-inflammatory cytokines [179, 180], which correlated with memory impairment [180] and general cognitive decline [181]. In older nondemented people, MRI studies demonstrated macrostructural brain abnormalities linked to inflammation, including reduced hippocampal and GM volume, global brain atrophy, cortical thinning, and WM hyperintensity, which may partly explain the age-related cognitive decline [182–184]. Recently, an association between reduced microstructural integrity of WM pathways and higher circulating inflammatory markers (i.e., C-reactive protein and tumor necrosis factor-alpha, TNF-*α*) was found in middle-aged and elderly people, which correlated with higher-order cognitive functions impairment [182]. Neuroinflammation can be a cause (by generating reactive oxygen and nitrogen species) or a consequence of chronic oxidative stress (OS). Over time, OS triggers a self-perpetuating cycle of chronic neuroinflammation inducing even more OS, leading to neuronal degeneration and cell death [185]. Finally, aging effects on gut microbiota may induce a higher propensity to develop the* Clostridium difficile* infection which enhances local and systemic proinflammatory markers (IL-1*β*, TNF-*α*, and CRP) and increases the permeability of gut barrier [186].

In conclusion, anxiety seems to be associated with activated immune-inflammatory pathways, which are also a characteristic of aging.

### 9.3. Mechanisms by Which Activated Immune-Inflammatory Pathways May Contribute to Accelerated Aging and Neuroprogression

**Figure 2:**
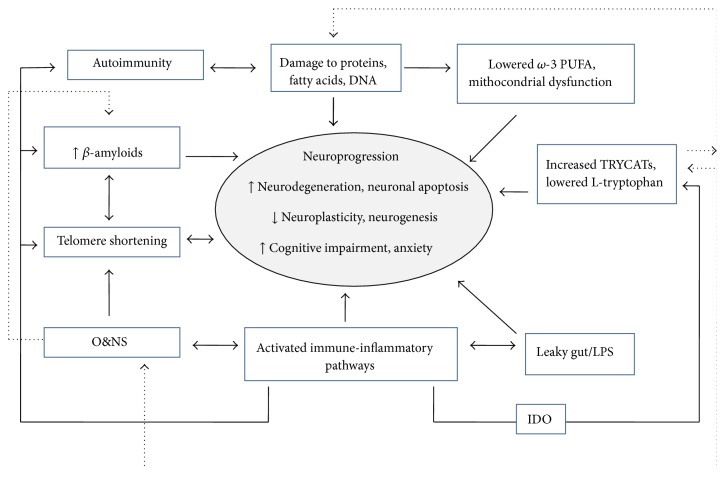
A global model of neuroprogressive changes in aging and anxiety. CMI and inflammatory responses are accompanied by activation of O&NS with production of increased ROS/RNS. ROS/RNS can react with proteins, fatty acids (including *ω*-3 PUFAs), and DNA and change their chemical structure which became immunogenic and produce an autoimmune response. Both O&NS and CMI inflammation are implicated in beta-amyloid formation and telomeres shortening. Moreover, increased O&NS impairs mitochondrial function with further production of ROS and macromolecular damage. PICs activate IDO which causes depletion of tryptophan/5-HT and the synthesis of tryptophan catabolites (TRYCATs). Some of these TRYCATs (kynurenine and quinolinic acid) are anxiogenic and neurotoxic. The lipopolysaccharide (LPS), caused by bacterial translocation from the gut, may aggravate existing inflammation and O&NS or trigger a primary inflammatory response. LPS and lowered *ω*3 are associated with decreased neurogenesis. CMI: cell-mediated immune; PICs: proinflammatory cytokines; IL-1*β*: interleukin-1*β*; IL-6: interleukin-6; TNF-*α*: tumor necrosis factor-*α*; IFN-*γ*: interferon-gamma; O&NS: oxidative and nitrosative stress; ROS: reactive oxygen species; RNS: reactive nitrogen species; IDO: indoleamine 2,3-dioxygenase; TRYCATs: L-tryptophan catabolites; LPS: lipopolysaccharide (a component of the outer membrane of Gram-negative bacteria); *ω*-3 PUFAs: omega-3 polyunsaturated fatty acids.

The immune-inflammatory pathways may contribute to accelerated aging and neuroprogression by several mechanisms. In rats, elevated IL-2 levels are associated with neurocognitive impairments, microglial activation, reactive astrogliosis, myelin damage, neuronal loss, and changes in several receptors, such as cholinergic and/or dopaminergic receptors in frontoparietal cortex and hippocampal regions [187, 188]. By inducing IDO activation, elevated IFN-*γ* may lower serotonin levels (5-HT) and increase TRYCATs, with negative effects on neuronal survival. Indeed, lower 5-HT may negatively affect neurogenesis and BDNF expression in adult mammals [189]. TRYCATs, especially quinolinic acid, may increase production of reactive oxygen species (ROS), induce mitochondrial dysfunctions, exert neurotoxic effects by acting as NMDA-receptor agonists, and cause hippocampal cell death and reduction in cerebral cholinergic circuits in rodents [190–192]. Recently, activation of the kynurenine pathway has been shown to affect hippocampal neurogenesis in humans [193]. Increased levels of IL-6 may have neurodegenerative effects in mice [194] and IL-1*β* may exert neurotoxic effects with neuronal death [195, 196], impair hippocampal neurogenesis [197], and reduce BDNF expression [198]. TNF-*α* may potentiate glutamate neurotoxicity and silence cell survival signals [199]. LPS can cause cell death by inducing apoptosis and increasing levels of ROS and reactive nitrogen species (RNS) [200]. Finally, activated immune-inflammatory pathways are implicated in A*β* formation [201], telomere shortening [202, 203], and increasing of O&NS [153] that, in turn, is highly implicated in aging and neuroprogression (see the following sections) (Figure 2).

## 10. Oxidative/Nitrosative Stress

Oxidative/nitrosative stress (O&NS) may come from free radicals (FR) (superoxide, hydroxyl radical) or nonradical molecules, like hydrogen peroxide, and their derivatives, that is, reactive oxygen species (ROS) and reactive nitrogen species (RNS). Inflammation and mitochondrial processes are sources of ROS and RNS. Under normal conditions, the potentially damaging effects of increased ROS and RNS are counterbalanced by enzymatic and nonenzymatic antioxidant defense systems [16]. Activation of O&NS occurs when excess of ROS/RNS and/or compromised antioxidant mechanisms are present. Consequently, O&NS may damage cellular structures such as DNA, lipids (including omega-3 PUFAs), proteins, mitochondria, and cell membranes, up to cellular death [204]. These processes alter the endogenous fatty acids and proteins and may render them immunogenic, inducing autoimmune responses against these modified antigenic determinants (neoepitopes) that lead to a vicious circle resulting in additional cell dysfunctions or death [205]. Finally, O&NS activates immune-inflammatory pathways [153] (Figure 1).

### 10.1. Oxidative/Nitrosative Stress in Anxiety

O&NS seems to be involved in the pathogenesis of AnxDs [206]. In murine models, several paradigms inducing distress and anxiety-like behaviors resulted in decreased activity of antioxidant enzymes with increased oxidative damage to lipids, proteins, and DNA in multiple brain areas, such as the hippocampus, prefrontal cortex, and cerebellum. Gene expression and proteomic studies in various mice models of anxiety also showed connections between high anxiety and dysregulated expression of several proteins related to oxidative stress metabolisms [207]. Direct induction of high oxidative stress in rats or knockout mice models induced increased anxiety-like behaviors [207, 208], while antioxidant treatments reduced both oxidative stress markers and anxiety-like behaviors [208]. Similarly, indirect induction of oxidative stress via acute sleep deprivation caused anxiety-like behaviors and memory impairment in rats [209]. In mice, deficiency of the antioxidant vitamin E increased oxidative stress and anxiogenic behaviors [210]. A diet rich in *ω*3 eicosapentaenoic acid (EPA) (an omega-3 PUFA that is located in cellular membranes, has anti-inflammatory properties, and is damaged by O&NS [211]) reduces the development of anxiety-like behaviors in rats as well as normalizing dopamine levels in their ventral striatum [212]. Changes in mitochondrial energy metabolism and function related to O&NS have been associated with anxiety in preclinical studies. In a trait anxiety mouse model, high anxiety-related behaviors were associated with mitochondrial dysfunction in cingulated cortex with enhanced oxidative stress lipid peroxidation and cell death [213]. Finally, anxiety-like behaviors exhibited by rodents during aging may be partly due to increased oxidative stress levels [214].

Findings in humans with AnxDs are mixed, probably due to methodological differences among studies and several confounding factors that may influence oxidative markers and pathways [207]. However, most studies supported the hypothesis of a connection between anxiety and increased oxidative stress. Individuals with lower serum *ω*3 or with a higher *ω*6/*ω*3 ratio (*ω*6 has proinflammatory effects) have significantly higher stress-induced anxiety levels and TNF-*α* and IFN-*γ* responses compared to those with higher serum *ω*3 and a lower *ω*6/*ω*3 ratio [211, 215]. In line with this, *ω*3 supplementation reduced inflammation and anxiety among healthy young adults who faced stressful major examination [216]. In patients with SAD [217, 218] and PD [219], increased levels of blood lipid peroxidation (a marker of oxidative stress-related cellular damage) were found. Adult subjects with PD [220], with PD, and with agoraphobia [221] and children and adolescents with AnxDs [222] exhibited impaired oxidative balance and higher oxidative stress. In patients with GAD, decreased levels of the antioxidant serum free sulphydryl were found, which negatively correlated with disease duration [223]. Finally, preliminary, non-placebo-controlled studies showed that selective serotonin reuptake inhibitors (SSRIs) treatment (the first-line drug therapy for AnxDs) decreased both oxidative stress and clinical symptoms in subjects with PD and SAD [217, 220], suggesting that oxidative stress may be a state condition related to the anxious symptomatology.

### 10.2. Oxidative/Nitrosative Stress in Aging

O&NS contributes consistently to aging. Animal studies showed that reducing oxidative stress/damage increased the healthy period of life [224–226], while a chronic deficit of the antioxidant vitamin C accelerated oxidative stress and amyloid deposition during normal aging [227]. In humans, oxidative stress has been associated with cognitive dysfunction during normal aging [228], in mood disorders [229, 230], and in schizophrenia [231]. The O&NS-induced damage of membrane *ω*3 is thought to be implicated in aging. Indeed, the *ω*3 eicosapentaenoic acid (EPA) has a protective effect on neurons [232] and experimental evidence indicated that *ω*3 docosahexaenoic acid- (DHA-) enriched diet can protect the brain from cognitive decline in aged rats [233]. In humans, a recent meta-analysis exploring the association between *ω*3 and risk of cognitive decline in elderly individuals has shown that daily doses from 400 to 1800 mg (for 3–40 months) may significantly decrease the cognitive decline [234].

Finally, oxidative damage to mitochondrial functions and macromolecules are thought to play key roles in aging processes. According to the mitochondrial theory of aging [235], ROS-induced mutations of mitochondrial DNA increase over the lifespan and alter mitochondrial respiratory function leading to further increasing of ROS and damage to DNA as well as to other macromolecules, up to irreversible cellular senescence.

In conclusion, anxiety seems to be associated with increased O&NS, which is also implicated in processes of aging.

### 10.3. Mechanisms by Which Oxidative/Nitrosative Stress May Contribute to Accelerated Aging and Neuroprogression

O&NS may contribute to accelerated aging and neuroprogression by multiple mechanisms. Damage by O&NS involves lipid peroxidation, oxidatively induced protein and DNA alterations, altered neuronal signaling, and neuronal apoptosis [236]. O&NS-induced lower *ω*3 PUFAs may be associated with decreased neurogenesis, since *ω*3 PUFAs have beneficial effects on serotonin metabolism stimulating neurogenesis, increase BDNF expression, and exert anti-inflammatory activity [232, 237]. O&NS processes may also cause damage to mitochondria, which play a central role in energy production (in form of adenosine triphosphatase, ATP), are involved in metabolism of amino acids, lipids, and steroids, and regulate free radicals' levels, intracellular calcium concentration, and processes implicated in synaptic development and cell death [238]. Mitochondrial dysfunction impairs neural progenitor cell function [239] and may affect several brain functions by decreasing ATP production. Indeed, high levels of energy are needed for brain activities, including synaptic remodelling, signal transduction, and maintenance of transmembrane potential [240], and deficiency of ATP may lead to activation of the apoptotic cell death program [241]. Disrupted mitochondrial function also provokes mitochondrial-derived hyperproduction of ROS that causes a self-perpetuating cycle of O&NS, inducing even more mitochondrial and macromolecule damage, up to neuronal degeneration and cell death [242]. Finally, O&NS activates immune-inflammatory pathways [153], can induce accelerated telomere shortening and reduce telomerase activity [243, 244], and may be implicated in A*β* formation [245, 246] (Figure 2).

## 11. Discussion

In this paper, we explored the association between AnxDs and hallmarks of accelerated aging, with a focus on neuroprogression. We reviewed animal and human findings that suggest an overlap between processes of impaired neurogenesis, neurodegeneration, structural, functional, molecular, and cellular modifications in AnxDs, and aging. A putative global model of neuroprogressive changes in aging and anxiety is summarized in Figure 2. Although several studies pointed to a model of accelerated aging and neuroprogression for depression and bipolar disorder [12, 16, 19, 247–249], this research on AnxDs is at an early stage and some caveats should be taken into account. First, since the available data are very limited, we considered AnxDs as a group and reported findings also from nonclinical populations. However, neurobiological mechanisms implicated in the different AnxDs or in subjects with anxiety symptoms but without full-blown disorders do not completely overlap, and AnxDs differ in their incidence across lifespan [3]. Future studies are needed to investigate the specific association of each anxiety disorder with accelerated aging and whether differences exist between clinical and nonclinical populations. Second, brain imaging studies on AnxDs yielded mixed findings due to sampling, methodology, and AnxDs heterogeneity. All the same, a detailed description of these inconsistencies was beyond the scope of this paper. In line with our aim, we only reported evidence common to both anxiety and aging. Third, available animal models of anxiety are based on different theoretical constructs and to date their translational validity is still debated [250]. Thus, parallelisms between animal and human studies should be considered with caution. Fourth, multiple genetic, environmental, and individual factors may influence the biological processes involved in aging and neuroprogression, such as immune-inflammatory pathways, O&NS, telomere shortening, and A*β* generation [149]. Since the role of these confounding factors has not been exhaustively investigated, the findings of an association between these processes and the AnxDs should be considered with prudence. Finally, most studies were cross-sectional; thus, it was not possible to clarify any causal path between anxiety and aging. Considering these limitations, our review suggests a link between anxiety and accelerated aging across multiple processes involved in neuroprogression. Several brain structural and functional changes that accompany normal aging were more pronounced in subjects with AnxDs than in coevals without AnxDs, including reduced GM density, WM alterations, impaired functional connectivity of large-scale brain networks (in particular the DMN), and poorer cognitive performance. Preliminary prospective findings suggested that, in older individuals, anxiety symptoms are risk factor for accelerated cognitive decline, independently of depression. Similarly, molecular correlates of brain aging, such as telomere shortening, A*β* accumulation, immune-inflammatory pathways, and O&NS, were overrepresented in anxious subjects compared with coeval nonanxious subjects, especially when anxiety was severe and long-lasting. These preliminary results do not allow drawing any conclusion about causality or directionality between anxiety and accelerated aging, and future longitudinal studies are needed to shed some light on this issue. Several scenarios are possible: for example, (1) AnxDs may accelerate age-related molecular processes resulting in precocious brain structural changes and functional decline; (2) age-related processes may lead to AnxDs over time; (3) aging and AnxDs may reciprocally influence each other and/or may share some genetic and/or environmental factors which may increase vulnerability to both AnxDs and accelerated aging with neuroprogression. According to the first hypothesis, the sustained arousal and neurobiological sensitivity to different threats in anxious subjects might cause the prolonged activation of HPA axis and ANS, which, in turn, may result in increased immune-inflammatory and oxidative/nitrosative stress (IO&NS) with a self-perpetuating chronic cycle leading to telomere shortening, precocious cellular aging, neurodegeneration, and impaired neuroplasticity [137, 251, 252]. On the contrary, according to the second hypothesis, age-related molecular changes and aging of the human brain may engage biological mechanisms similar to those implicated in anxiety, such as dysregulation of HPA axis, increased IO&NS, and impaired limbic-frontal areas connectivity. Thus, age-related processes, in combination with environmental/genetic factors, may promote the development of at least some AnxDs in vulnerable individuals [253]. This hypothesis fits with the idea that AnxDs may be neurodevelopmental disorders occurring at different lifespan stages and with the higher prevalence of some AnxDs, such as GAD, in adulthood and older age [3]. Finally, according to the third hypothesis, mutual amplifications are likely implicated in the biological processes of anxiety and aging. Indeed, both conditions are accompanied by activation of IO&NS pathways, which exhibit reciprocal reinforcement and, in turn, contribute to telomere shortening, accelerated aging, and neuroprogression [203, 207, 254]. Immune-inflammatory pathways are involved in both anxiety and cognitive decline, also by stimulating the HPA-axis function with cortisol release that modulates anxiety behavior and exerts detrimental effects on cognition [255].

In conclusion, preliminary evidence indicated an association between AnxDs and hallmarks of accelerated aging with phenomena of neuroprogression. Withal additional animal and human research is needed to satisfactorily elucidate these questions.

### 11.1. Implications for Treatment and Future Research

AnxDs are complex diseases which tend to be chronic when not adequately treated. Unfortunately, even evidence-based treatments, such as cognitive-behavioral therapy and SSRIs, are often not able to produce full remission and the rate of relapses after drug discontinuation is significant [256]. The theoretical framework of an association between accelerated senescence, neuroprogression, and anxiety may suggest some implications and strategies to fill these gaps. In addition to clinical symptoms of AnxDs, the use of biomarkers (such as inflammatory, oxidative, and telomere length markers) and cognitive assessment may help to better characterize the patients' profiles and clinical stages and allow more personalized treatments. The modifications of these markers during treatments may render the treatments more efficacious and represent reliable treatment-outcome predictors. Moreover, treatments specifically targeting these mechanisms, including both pharmacological and nonpharmacological “antiaging” interventions, may increase the rate of favorable outcomes. Indeed, preclinical studies suggested that some drugs currently used for AnxDs normalize some hallmarks of accelerated aging and exert a neuroprotective effect. In mice, alprazolam, zolpidem, and buspirone ameliorated the oxidant/antioxidant balance decreasing nitrite concentration and lipid peroxidation in the brain [257] and the SSRI fluoxetine reversed the decreased activity of telomerase in the hippocampus induced by chronic mild stress [258]. SSRIs promoted synaptic plasticity and neurogenesis in mice, probably by increasing BDNF, improved spatial memory learning [259], and facilitated learning and memory during aging [260]. In rats, treatments with antioxidants reduced both oxidative stress and anxiety-like behaviors [261] and in older animals increased serotonin levels [262]. In humans, preliminary data showed that SSRIs reversed high oxidative stress in patients with depression, PD, or SAD [217, 220, 263], promoted hippocampal neurogenesis [264] in depressed subjects, and decreased A*β* production in the cerebrospinal fluid of healthy individuals [265]. Finally, successful pharmacological treatment with the SSRI escitalopram in late-life GAD was associated with episodic memory and executive functioning improvement [90]. Nonpharmacological treatments may include physical activity and nutritional interventions. In mice, physical activity increased telomerase activity and cognitive performance [266, 267] and decreased both oxidative stress and anxiety-like behaviors [208]. In humans, physical activity increased brain volume [268] and preserved cognitive functions in healthy older adults [269], improved comorbid anxiety and executive functioning impairment [270], and has been proposed as a neuroprotective strategy with antioxidant properties [271]. Recent investigations suggested that Mediterranean dietary pattern slowed cognitive decline and improved cognitive performance [272, 273] by reducing inflammation markers [274] and oxidative damage [275]. Higher intake of processed and unhealthy foods was associated with increased anxiety in a population-based study [276], while a healthier dietary pattern was associated with a reduced likelihood of anxiety or depressive disorders [277]. Finally, improvement in both cognition and anxiety was exerted by resveratrol, a component of grapes with important antioxidant properties [278]. Future studies should investigate whether treatments with “antiaging” properties may be beneficial to patients with AnxDs with hallmarks of accelerated aging and neuroprogression.
